# Neuromorphic Vision Datasets for Pedestrian Detection, Action Recognition, and Fall Detection

**DOI:** 10.3389/fnbot.2019.00038

**Published:** 2019-06-18

**Authors:** Shu Miao, Guang Chen, Xiangyu Ning, Yang Zi, Kejia Ren, Zhenshan Bing, Alois Knoll

**Affiliations:** ^1^College of Automotive Engineering, Tongji University, Shanghai, China; ^2^Robotics, Artificial Intelligence and Real-Time Systems, Technische Universität München, München, Germany

**Keywords:** dynamic vision sensor, dataset, pedestrian detection, action recognition, fall detection

## 1. Introduction

Large-scale public datasets are vital for algorithm development in the computer vision field. Thanks to the availability of advanced sensors such as cameras, Lidar and Kinect, massive well-designed datasets created by researchers are free to the scientific and academic world. ImageNet (Deng et al., 2009) is one of the most representative examples which is widely used for image recognition tasks in computer vision. UCF 101 (Soomro et al., 2012) is another large-scale dataset used for human action recognition. However, both of the above datasets provide only the appearance information of objects in the scene. With the limited information provided by RGB images, it is extremely difficult to solve certain problems such as the partition of the foreground and background which have similar colors and textures. With the release of the low-cost Kinect sensor in 2010, acquisition of RGB and depth data became cheaper and easier. Not surprisingly, increasing RGB-D datasets, recorded by the Kinect sensor and dedicated to a wide range of applications, have become available (Cai et al., 2017). We see the same trend, the KITTI dataset (Geiger et al., 2013), starting to occur in the autonomous driving community due to the availability of the Velodyne HDL-64E rotating 3D laser scanner. It is clear that the advent of new sensors always brings opportunities for new dataset development. In this data report, we introduce three new neuromorphic vision datasets recorded by a novel neuromorphic vision sensor named Dynamic Vision Sensors (DVS) (Lichtsteiner et al., 2008).

DVS is a novel type of neuromorphic-based vision sensor, developed by Lichtsteiner et al. (2008). The sensor records event streams as a sequence of tuples [*t, x, y, p*], where *t* is the timestamp of the event, (*x, y*) is the pixel coordinates of the event in 2D space and *p* is the polarity of the event indicating the brightness change. Compared to the conventional frame-based cameras, neuromorphic vision sensors are frameless which take a radically different approach, doing away with images completely. It properly addresses the universal drawbacks of conventional frame-based cameras, such as data redundancy, high latency and low temporal resolution in a fresh new paradigm. This sensor has matured to the point of entering commercial market only in the last decade. As a much younger field, one of the main challenges faced is the lack of neuromorphic vision datasets impeding the progress of the field. We can thus learn from the rapid development and maturation of computer vision.

It cannot be doubted that neuromorphic vision research will benefit from new datasets similar to those of computer vision. However, the unique difficulty in the datasets arises because neuromorphic vision data differs significantly from conventional camera data and no direct method for converting between two data formats exists. To address this, we introduce the largest neuromorphic vision datasets targeting the three human motion related tasks: pedestrian detection, human action recognition and human fall detection. We hope that those datasets will meet the significant demands of the neuromorphic vision, computer vision and robotic communities. More specifically, the open access of three datasets should stimulate the development of algorithms processing the event-based asynchronous stream input. In addition, to allow for a fair comparison with frame-based computer vision, we also introduce three encoding methods which are used to convert the spatio-temporal data format to conventional frames.

Previously, several datasets of neuromorphic vision sensors addressing the problem of detection and classification were proposed (Orchard et al., 2015; Serrano-Gotarredona and Linares-Barranco, 2015; Hu et al., 2016; Liu et al., 2016; Li et al., 2017). Many of them were recorded with a static DVS facing a monitor on which computer vision datasets were set to play automatically (Serrano-Gotarredona and Linares-Barranco, 2015; Hu et al., 2016). Thus, the intrinsic temporal information of moving objects between two frames are lost. It is gratifying that there are several high-quality datasets recorded in a real environment in recent years (Moeys et al., 2016; Zhu et al., 2018). Other pioneering works from iniLabs^1^ and the RPG group^2^. DDD17 dataset (Binas et al., 2017) is the first annotated driving dataset for event-format data. End-to-end prediction for the steering angle of a vehicle can be achieved with a convolutional neural network. Dataset for Pose Estimation, Visual Odometry, and SLAM is published by Mueggler et al. (2017b).

It is noteworthy that although there are many public datasets released by the neuromorphic vision community^3^, open-access datasets for human motion analysis are still lacking. Therefore, we aim to fill this gap and to introduce three datasets in this report: the pedestrian detection dataset, the action recognition dataset and the fall detection dataset. A DAVIS346redColor sensor^4^ is used for recording. Alongside the datasets, this report presents three encoding methods considering the frequency of the event (Chen, 2018), the surface of active events (Mueggler et al., 2017a) and the Leaky Integrate and Fire (LIF) neuro model (Burkitt, 2006), respectively. We conclude this report with the recording details and summaries of the datasets and encoding methods.

## 2. Materials and Methods

In this section, we first introduce the recording setup of those datasets. Further, specific recording procedures are shown. Three encoding approaches are finally provided.

### 2.1. Dataset Recording Setup

Those datasets are recorded with a DAVIS346redColor, a camera tripod and a laptop. DAVIS346redColor with a resolution of 346 × 260 is used to record real-world scenes. For each event [*t, x, y, p*], *x* ∈ [0, 345] and *y* ∈ [0, 259]. In order to reduce data storage size, APS frames were not recorded. The spatio-temporal event data in *aedat* format was saved with jAER software^5^.

### 2.2. Recording Procedure

**Figure 1:**
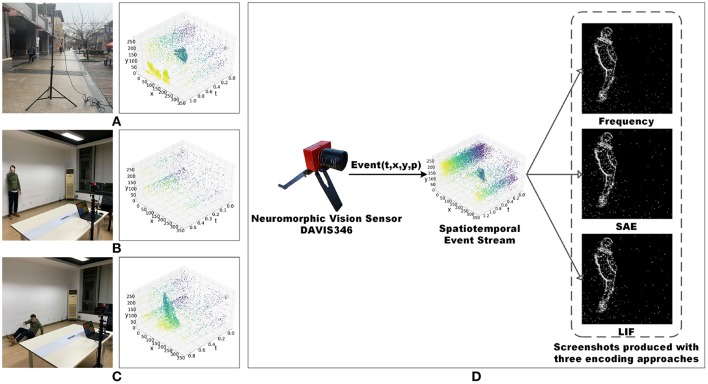
**(A)** Experiment environment setup of pedestrian recordings and corresponding data visualization. **(B)** Experiment environment setup of human action recordings and corresponding data visualization. **(C)** Experiment environment setup of human fall recordings and corresponding data visualization. **(D)** Encoding process of three methods as Frequency, SAE and LIF. The corresponding produced screenshots are from the pedestrian dataset. We clarify that the human subject's face in the images is obscured, and the subject has agreed on the publication of this figure.

We used a retractable tripod with a maximum elongation of five meters and a two-axis gimbal to make the field of view cover the entire region of interest. The pedestrian detection dataset was recorded in both indoor and outdoor scenarios, as shown in Figure 1A. The action recognition dataset and fall detection dataset were recorded in an empty office, as shown in Figures 1B,C.

### 2.3. Encoding Approaches

Standard computer vision algorithms cannot be used directly to process event data (Tan et al., 2015; Iyer et al., 2018). To address this problem, we introduce three encoding approaches here as *Frequency* (Chen, 2018), *SAE (Surface of Active Events)* (Mueggler et al., 2017b) and *LIF, (Leaky Integrate-and-Fire)* (Burkitt, 2006) to convert the asynchronous event stream to frames (Chen et al., 2019). The event data encoding procedure is shown in Figure 1D.

#### 2.3.1. Frequency

Given that many more events would occur near the object edges, we utilized the event frequency as the pixel value to strengthen the profile of the object. At the same time, noise caused by the sensor could be significantly filtered out due to its low occurrence frequency at a particular pixel within a given time interval. Concretely, we counted the event occurrence at each pixel (*x, y*) for accumulating each event into frames, based on which we calculated the pixel value using the following range normalization equation inspired by Chen (2018):

(1) $$\begin{array}{l} \sigma \left(n\right)=255\cdot 2\cdot \left(\frac{1}{1+e^{-n}}-0.5\right)\; \end{array}$$

where *n* is the total number of the occurred events (*positive* *or* *negative*) at pixel (*x, y*) within a given interval, and σ(*n*) is the value of this pixel in the event frame, the range of which is normalized between 0 and 255 in order to fit a 8-bit image.

#### 2.3.2. Surface of Active Events (SAE)

In order to take full advantage of the unique characteristic that neuromorphic vision sensors can record the exact occurring time of incoming events with low latency, the *SAE (Surface of Active Events)* (Mueggler et al., 2017b) approach is applied to reflect time information while the pixel value and its gradient can tell the moving direction and speed of the event stream. Specifically, regardless of the event polarity, each incoming event [*t, x, y, p*] will change the pixel value *t*_*p*_ at (*x, y*) according to the timestamp *t*. In this way, an grayscale image frame is acquired according to the timestamp of the most recent event at each pixel:

(2) $$\begin{array}{l} SAE:t\Rightarrow t_{p}\left(x,y\right)\; \end{array}$$

Moreover, to attain an 8-bit single channel image, numerical mapping is conducted by calculating the Δ*t* between the pixel value *t*_*p*_ and the initial time *t*_0_ for each frame interval *T* as follows:

(3) $$\begin{array}{l} g\left(x,y\right)=255\cdot \frac{t_{p}-t_{0}}{T}\; \end{array}$$

#### 2.3.3. Leaky Integrate-and-Fire (LIF)

According to the *LIF (Leaky Integrate-and-Fire)* neuron model (Burkitt, 2006), we regard every image pixel (*x, y*) as a neuron with its Membrane Potential (MP) and firing counter *n*. The MP value of a neuron will be influenced by input spikes and time-lapse. In detail, each incoming event at pixel (*x, y*), regardless of polarity, will trigger a step increase of this pixel's MP value. Simultaneously, MP value of each pixel will decay at a fixed rate. When the MP value of a pixel exceeds the preset threshold which is chosen based on the effect of LIF output, a firing spike output will be generated there, and the MP value of this pixel will be reset to 0 with no latency.

In a specific time interval, we count the number of the firing spike outputs for each pixel, i.e., the occurrence of events (recorded as firing counter *n*). After each interval, the firing spikes counter *n* of each pixel will be reset to 0. The accumulated grayscale frame can thus be obtained.

#### 2.3.4. Summary

Three different event-stream encoding methods are presented according to their ability to reflect different aspects of the event information. For the *Frequence* encoding method, the edges of the object will be strengthened to a great extent, which is beneficial for object detection as we have a clearer profile of the object. For the *SAE* encoding method, the raw timestamp information is directly utilized while the pixel value and its gradient can tell the moving direction and speed of the event stream. For the *LIF* encoding method, historical event data have been considered so that the output frames contain more past information. Three encoding methods can be used independently or as a fusion.

## 3. Results

We provide three neuromorphic vision datasets for pedestrian detection, human action recognition and fall detection, respectively. All the recordings, annotation files and source code of the three encoding methods are free to the public via this website^6^. The characteristics of three datasets are summarized in Table 1. Details are provided below.

**Table 1:** Characteristics of the three provided benchmark datasets.

	**Pedestrian detection dataset**	**Action recognition dataset**	**Fall detection dataset**
Number of subjects	–	15	15
Number of recordings	12	450	180
Average length	30 s	5 s	5 s
Number of labeled frames	4670	–	–
Scenarios	Corridor, walking street and square	Office	Office
Weather	Sunny, Rainy	–	–
Sensor		DAVIS346redColor	
Resolution	346 × 260	346 × 260	346 × 260
Number of classes	-	10, including arm-crossing, getting-up, kicking, picking-up, jumping, sitting-down, throwing, turning-around, walking and waving	4, including falling, bending, slumping-down, tying-shoes

### 3.1. Pedestrian Detection Dataset Recordings

**Figure 2:**
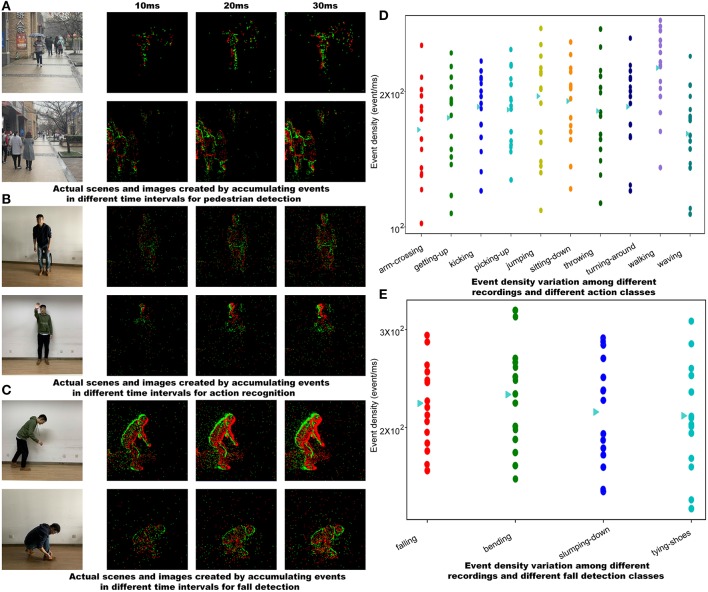
**(A)** Actual scenes and pre-processed frames of pedestrian. **(B)** Actual scenes and pre-processed frames of human action. **(C)** Actual scenes and pre-processed frames of fall. **(D)** Event density variation among different recordings and different action classes. **(E)** Event density variation among different recordings and different fall detection classes. Each point in figures **(D,E)** corresponds to all recordings from one subject. The triangles plot the overall mean for each action or fall detection class. All the faces of the subjects shown in this figure have been obscured.

The pedestrian dataset is set to record many scenarios, such as *corridor, walking street*, and *square*. All recordings last about 30 s, with slight variations in length. Each recording lasts about 30 s, in which multiple scenarios that are commonly seen in traffic surveillance tasks such as pedestrian overlapping, occlusion and collision are contained. Figure 2A shows an example from pedestrian detection recordings. A large part of raw data is converted to 4,670 frame images through a *SAE* encoding method with a time interval of 20*ms*. which equals to 50 fps of a conventional frame-based sensor. All these images have been labeled via annotation tool labelImg^7^.

### 3.2. Action Recognition Dataset Recordings

The action recognition dataset is recorded in an empty office, with 15 subjects acting out 10 different actions. Each subject shall perform three times for each pre-defined action within 5 s, and the camera is set to three positions and from different distances to the subject for recording each action. Recorded files are named after each action as *arm-crossing, getting-up, kicking, picking-up, jumping, sitting-down, throwing, turning-around, walking*, and *waving*. Figure 2B shows a recording for the sequence of jumping. The event density analysis, which shows motion variation among subjects, is presented in Figure 2D.

### 3.3. Fall Detection Dataset Recordings

The fall detection dataset is recorded with 15 subjects in an empty office. Actions are pre-defined as *falling, bending, slumping down* and *tying-shoes*, in which *falling* is a positive sample and the rest are negative samples. Each subject performs each action within 5 s and repeats it three times. The position of the camera changes according to the subject. Figure 2C shows sample recordings for the fall sequence. Figure 2E presents the event density analysis.

## 4. Discussion

We presented three neuromorphic vision datasets for pedestrian detection, human action recognition and human fall detection with DAVIS346redColor, which are freely available at Github. These datasets contain 642 recordings in jAER (.aedat) format and 4,670 annotated frames converted from event streams of pedestrian detection. To make event data for training fit with conventional neural networks, three different event-stream encoding approaches are provided with an open-source code. It is worth noting that the neuromorphic vision sensor is a perfect sensor to solve the privacy problems which always occurs for traditional frame-based cameras (e.g., pedestrian's face may be recognized in public computer vision dataset such as ImageNet). The raw data of the DVS sensor are event streams which only keep the shapes and movements of the subjects. In other words, there is no texture or appearance information recorded in our dataset. Therefore, it is impossible to identify subjects from our dataset which highlights one of the advantages of neuromorphic vision sensor over traditional frame-based cameras. We hope that these datasets can contribute to the advance of algorithms for neuromorphic vision sensor data, and further boost the development of neuromorphic vision.

It is noted that a fraction of recordings for pedestrian detection are spotted with dense noisy events caused by raindrops. And illumination changes outside will result in tiny noisy events on the recordings. These phenomena indicate that appropriate approaches for filtering event data are supposed to be adopted according to the purpose of researchers. However, details in this data report as well as images shown above prove that the datasets presented here are of high fidelity and high quality, which benefit from low latency and high temporal revolution of DVS.

## Data Availability

The datasets released in this study are available in https://github.com/MSZTY/PAFBenchmark.

## Author Contributions

SM, GC, XN, YZ, and KR recorded the data. GC and ZB wrote the paper. AK designed the structure of this data report.

### Conflict of Interest Statement

The authors declare that the research was conducted in the absence of any commercial or financial relationships that could be construed as a potential conflict of interest.

## Footnotes

^1^https://inilabs.com/

^2^http://rpg.ifi.uzh.ch/index.html

^3^https://github.com/uzh-rpg

^4^https://inivation.com/support/hardware/davis346/

^5^https://github.com/SensorsINI/jaer/releases

^6^https://github.com/MSZTY/PAFBenchmark

^7^https://github.com/tzutalin/labelImg/releases
